# A Modified Surgical Ventricular Reconstruction in Post-infarction Mice Persistently Alleviates Heart Failure and Improves Cardiac Regeneration

**DOI:** 10.3389/fcvm.2021.789493

**Published:** 2021-12-24

**Authors:** Siyuan Ma, Junyu Yan, Dexuan Yang, Wangjun Liao, Jianping Bin, Hairuo Lin, Yulin Liao

**Affiliations:** ^1^Department of Cardiology, State Key Laboratory of Organ Failure Research, Guangdong Provincial Key Laboratory of Shock and Microcirculation, National Clinical Research Center of Kidney Disease, Guangdong Provincial Institute of Nephrology, Nanfang Hospital, Southern Medical University, Guangzhou, China; ^2^Department of Oncology, Nanfang Hospital, Southern Medical University, Guangzhou, China

**Keywords:** surgical ventricular reconstruction, myocardial infarction, ventricular aneurysm, heart failure, myocardial regeneration

## Abstract

**Objectives:** Large ventricular aneurysm secondary to myocardial infarction (MI) results in severe heart failure (HF) and limits the effectiveness of regeneration therapy, which can be improved by surgical ventricular reconstruction (SVR). However, the conventional SVR procedures do not yield optimal long-term outcome in post-MI rodents. We hypothesized that a modified SVR procedure without aggressive purse string suture would persistently alleviate HF and improve cardiac regeneration in post-MI mice.

**Methods:** Adult male C57 mice were subjected to MI or sham surgery. Four weeks later, mice with MI underwent SVR or 2nd open-chest operation alone. SVR was performed by plicating the aneurysm with a single diagonal linear suture from the upper left ventricle (LV) to the right side of the apex. Cardiac remodeling, heart function and myocardial regeneration were evaluated.

**Results:** Three weeks after SVR, the scar area, LV volume, and heart weight/body weight ratio were significantly smaller, while LV ejection fraction, the maximum rising and descending rates of LV pressure, LV contractility and global myocardial strain were significantly higher in SVR group than in SVR-control group. The inhibitory effects of SVR on LV remodeling and HF persisted for at least eight-week. SVR group exhibited improved cardiac regeneration, as reflected by more Ki67-, Aurora B- and PH3-positive cardiomyocytes and a higher vessel density around the plication area of the infarcted LV.

**Conclusions:** SVR with a single linear suture results in a significant and sustained reduction in LV volume and improvement in both LV systolic and diastolic function as well as cardiac regeneration.

## Introduction

Heart failure (HF) secondary to myocardial infarction (MI) remains one of the most difficult clinical challenges worldwide despite the continuous development of new drugs and interventional therapies (1). Current evidence suggests that prompt optimal medical therapy and revascularization after MI can reduce the incidence of HF, but a certain proportion of patients with MI inevitably develop into HF, especially patients with left ventricular aneurysm (LVA) and a larger left ventricular (LV) volume (2). LV remodeling is a critical step in the progression to HF in MI patients, and reversion of LV remodeling can alleviate or even cure HF (3, 4). However, medical and interventional therapies are insufficient to reverse the severe remodeling in some patients with refractory HF (5), which requires the use of more powerful techniques such as surgical correction to improve the worse prognosis.

Surgical ventricular reconstruction (SVR) has been used to reverse LV remodeling and ameliorate HF for more than half a century (6). In 2004, the RESTORE team reported that SVR after coronary artery bypass grafting alleviated HF and was an effective therapy for ischemic cardiomyopathy, showing excellent five-year outcomes (5). In contrast, in 2009, the STICH trial showed that SVR did not result in greater benefits than coronary artery bypass grafting alone (5, 7). Interestingly, a minimally invasive hybrid procedure called the Revivent TC system is emerging as a new treatment approach for MI patients with LVA, an enlarged LV and HF, and the results are encouraging thus far (8, 9). The current concerns related to conventional SVR include its impacts on LV diastolic function and right ventricular function, as well as the long-term outcome (10, 11). Basic research using ideal animal models would provide valuable clues and therapeutic strategies to address these concerns.

Animal experiments can elucidate the mechanism underlying clinical problems due to their good homogeneity. To date, animal studies on SVR have been limited. It was reported that the initial effects of LV repair on LVA in post-MI rats were not long-lasting, but could be extended by adjuvant use of ACE-I (12). Recently, an SVR rat model was used as an improved tool to test the efficiency of cardiac regeneration therapy (13, 14). Clinical findings suggest that improved operation procedures would result in better outcomes. Previous SVR rodent models generated by plicating the LVA with an aggressive purse string suture commonly exhibit postoperative recurrent LV dilation and deterioration of HF (12, 15).

Considering that mouse is an ideal model animal for molecular biology studies and genetic manipulation and that 100% of mice with MI develop LVA, an increased LV volume and severe HF, it is reasonable to generate an SVR mouse model without recurrent LV dilation. We hypothesize that a proper SVR procedure could persistently alleviate HF and increase cardiac regeneration in mice after MI.

## Materials and Methods

All procedures were carried out in conformity with the guidelines for animal research of our institutional, which conform to the Guide for the Care and Use of Laboratory Animals (National Institutes of Health Publication 8th Edition, 2011). This study was approved by the Ethical Committee of Nanfang Hospital, Southern Medical University (Guangzhou, China).

### MI and SVR Mouse Model

To generate MI model, male C57BL/6 mice (8–10 weeks age, 20–25 g weight) were anesthetized with a mixture of xylazine (5 mg/kg) and ketamine (100 mg/kg) through intraperitoneal injection, and the left thoracotomy and left coronary artery ligation were performed as described elsewhere (16). Successful ligation was confirmed by both ST-segment elevation on the electrocardiogram and paleness of the myocardium. The infarct size was determined by triphenyltetrazolium chloride (TTC) staining at 6 h after surgery (17). Four weeks after MI, half of the mice with MI and an LV end-systolic volume index (LVESVI) >12 ml/m^2^ were randomly selected for SVR.

#### SVR Procedure

Mice were anesthetized with a mixture of xylazine (5 mg/kg) and ketamine (100 mg/kg) by intraperitoneal injection and intubated as described elsewhere (17). A 1.5 cm incision was made in the skin below the suture scar, and the intercostal muscle was cut between the 4th and 5th ribs to expose the heart. After careful confirmation of the aneurysm boundary, a single suture was performed with a 6-0 nylon thread from the upper left of the ligation knot remaining from MI surgery to the right side of the cardiac apex, and the surgical knot was used to fold the aneurysm without puncturing it. Unlike in conventional procedures, neither purse string suture nor aneurysm resection was performed. Control mice underwent a 2nd thoracotomy only. The thoracic cavity was closed layer by layer with 5-0 nylon sutures. Detailed method of the SVR procedure is shown in Supplementary Video 1.

### Echocardiography and Measurement of Invasive LV Hemodynamic

Four weeks after MI or sham surgery, echocardiography was performed over a time course of 3 weeks (8 weeks for some mice) to assess the cardiac remodeling and function. Mice were anesthetized by inhaling 3% isoflurane and maintained anesthesia with 1.5–2% isoflurane by using a Vevo 2,100 system with a 30 MHz probe (Fujifilm VisualSonics, Ontario, Canada). During the operating process, mice were placed on the warming platform in the supine position and their heart rate was maintained between 400 and 550 beats/min (18, 19). M-mode images were recorded in the long-axis view of the LV to observe the cardiac morphology, and the two-dimensional parasternal short-axis views of LV were obtained at the papillary muscle level to guide the M-mode measurement of the LV diastolic posterior wall thickness (LVPWd, mm), LV systolic posterior wall thickness (LVPWs, mm), LV end-diastolic diameter (LVEDd, mm) and LV end-systolic diameter (LVEDs, mm). The Teichholz formula [V = 7D^3^/(2.4+D), V = volume, D = the internal diastolic or systolic dimension measured by echocardiography] was used to calculate the LV end-diastolic volume (LVEDV, ul) and LV end-systolic volume (LVESV, ul). The body surface area (BSA, m^2^) was calculated according to the Meeh-Rubner formula: BSA = k × (W^2/3^)/10,000, k = 9.1, W = body weight (BW, g). LV ejection fraction (LVEF, %), LV end-diastolic volume index (LVEDVI, ml/m^2^) and LV end-systolic volume index (LVESVI, ml/m^2^) were calculated as follows: LVEF = ((LVEDV-LVESV)/LVEDV) × 100%, LVEDVI = (LVEDV/1,000)/BSA, LVESVI = (LVESV/1,000)/BSA. The right ventricle (RV) parameters were obtained as described by Wang et al. and the four-chamber view was obtained to measure the tricuspid annular plane systolic excursion of tricuspid valve (TAPSE) as described elsewhere (20–22).

At 3 weeks after SVR, speckle tracking was performed in sham, MI, and MI+SVR group. The two-dimensional (B-mode) parasternal long axis and short axis echocardiography were reserved for VEVO Lab (Fujifilm Visual Sonics). In short, three consecutive cardiac cycles with obvious R wave under B-mode ultrasound were selected for analysis. The endocardial and epicardial boundary were manually circled and the related parameters of endocardium and epicardium were measured both in longitudinal and circumferential axial plane. The measurement indexes included: left ventricular area change fraction (FAC), left ventricular global longitudinal strain peak (GLS), global radial strain peak (GRS) and global circumferential strain peak (GCS) (23).

LV hemodynamics were measured by using a Millar catheter system before the mice were sacrificed as described elsewhere (19, 24). To ensure the accuracy of the hemodynamic data as much as possible, we standardized the anesthetic dose of the mice to 2/3 of the normal anesthetic dose based on body weight (BW). During the procedure, the mice were placed on a 37°C thermostat plate to ensure a consistent body temperature for all mice. All mice had no major bleeding or fluid loss during the invasive assay and were not given additional rehydration.

### Histology

Seven weeks after the 1st surgery, the mice were sacrificed by overdosing pentobarbital (150 mg/kg, i.p). BW, heart weight (HW) and tibia length (TL) were measured. The heart was fixed in 4% paraformaldehyde and embedded in paraffin according to standard protocols. Azan-Masson staining was used to evaluate myocardial fibrosis (25). Immunofluorescence staining was performed to evaluate the regeneration of cardiomyocytes (CMs) and vessels. Three to five different slides of the same sample were observed and averaged. Paraffin-embedded blocks of heart tissue waxes were cut into 4 mm tissue slices and then deparaffinized and subjected to antigen retrieval. Tissue sections were then rinsed three times with PBS for 5 min each and permeabilized for 20–25 min with 0.1% Triton X-100. After permeabilization, samples were rinsed three times with PBS for 5 min each and blocked with 2% bovine serum albumin (BSA) for 1 h at room temperature. Then, samples were incubated for 16–18 h at 4°C with primary antibodies [anti-Ki67 (1:600, Abcam, USA), anti-Ph3 (1:600, Invitrogen, USA), anti-Aurora B (1:600, Abcam, USA), anti-cTnI (1:200, Proteintech, USA), anti-αSMA (1:200, Abcam, USA)]. After incubation, samples were rinsed three times with PBS for 5 min each and stained with secondary antibodies (Alexa Fluor 488, Abcam, 1:200; Alexa Fluor 555, Abcam, 1:200) for 1 h at room temperature. DAPI sealing solution (Beyotime, USA) was used to stain the nuclear and seal the slices.

### RNA Quantification

Total RNA was obtained from mouse heart tissue with a total RNA isolation system (Omega, Norcross, GA, USA) and then converted to cDNA using oligo (dT) primers with PrimeScriptTM RT Master Mix (Takara Bio Inc., Shiga, Japan). Quantitative reverse transcription–polymerase chain reaction (qRT-PCR) was performed by using SYBR Green PCR Master Mix (Takara Bio Inc., Shiga, Japan) and a Light Cycler 480 System (Roche, Germany). The sequences of the primers are listed in Supplementary Table 1.

### Allocation of Sample Size for Mouse Experiments

A total of 140 mice were used in this study, of which 40 were used in the sham group and 100 were used for MI surgery at the beginning. Seventy-five mice survived after MI surgery. Sixty-two were eligible for SVR surgery after echocardiography certification. Of these 62 mice, five were randomly selected for sampling and PCR experiments. Then, 30 mice were randomly selected for SVR surgery (MI+SVR group) and the remaining 27 mice were kept as MI group. Five mice died during SVR surgery due to mislabeling or excessive blood loss. Ten mice in each group were selected for continuous observation of echocardiographic indices and statistics, and the remaining mice were measured at individual time points to ensure successful surgery. Three weeks after SVR, 6 sham mice, 7 MI mice and 5 SVR mice were randomly selected for whole transcriptome sequencing to better investigate the molecular biological mechanism of SVR (not involved in this study at this time, data are not shown), while 5 SVR mice were kept under observation until 8 weeks after SVR surgery, and the hearts of these mice were not stained or subjected to PCR experiments. For the remaining mice, 10 of each group were randomly selected for speckle tracking measurements, 5 mice for invasive hemodynamic testing, 10 mice for HW and TL measurements, 5 mice for histological staining (while 10 MI mice for Masson staining), and 3 to 5 mice for PCR of target genes in myocardial tissue.

### Statistical Analysis

The data were analyzed with GraphPad Prism 7.0 software (GraphPad Software, Inc., CA, USA). All quantitative data are presented as the mean ± standard error of the mean (SEM). Comparisons among multiple groups were performed using one-way analysis of variance followed by Bonferroni's correction or paired Student's *t*-test for multiple *post hoc* comparisons. Survival analysis was performed using the log-rank (Mantel-Cox) test. A *P*-value < 0.05 was considered statistically significant.

## Results

### SVR Reverses LV Remodeling in Mice After MI

**Figure 1:**
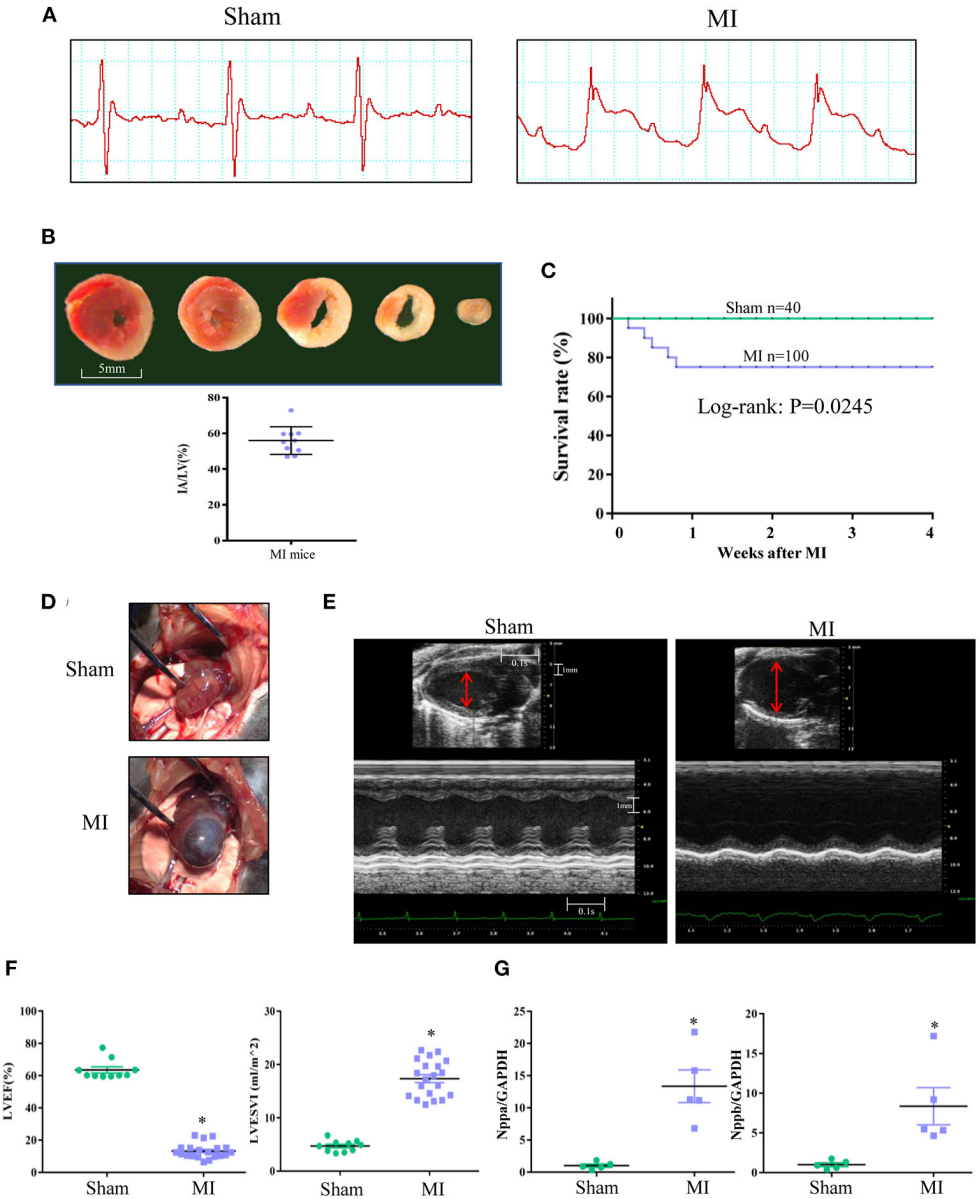
Mouse MI model characterized by serious heart failure with enlarged left ventricle (LV) and aneurysm. **(A)** Representative electrocardiogram showing classical ST-segment elevation in Lead II in response to left coronary artery ligation. **(B)** Infarct area (IA) determined by triphenyltetrazolium chloride (TTC) staining at 6 h after MI. Red staining area indicates survival myocardium, pale area indicates infarct area. Scale bar = 5 mm. **(C)** Mortality in the 1st week after MI and the survived MI mice after 1 week could survive to 4 weeks. **(D)** Representative image of LV aneurysm at 4 weeks after MI surgery (100% incidence). **(E)** Representative recordings of 2D and M-mode echocardiographic images of sham and MI mice at 4 weeks after surgery. Normal sham LV and enlarged LV of MI heart was indicated (red line). **(F)** LV ejection fraction (LVEF) and LV end systolic volume index (LVESVI) at 4 weeks after surgery. **(G)** Real-time PCR for myocardial natriuretic peptide type A (Nppa) and natriuretic peptide type B (Nppb) expression at 4 weeks after surgery. *P < 0.05 MI group vs. sham group. MI, myocardial infarction; Data are means ± SEM.

To induce sufficient LV remodeling, the mouse left coronary artery was ligated 1 mm below the left atrium, and a significant ST segment elevation and infarct size larger than 40% were used as quality control criteria (Figures 1A,B). Four weeks after MI, 75% of mice survived and exhibited severe LV remodeling, as manifested by a 100% incidence of LVA, a significantly increased LV volume, a markedly reduced LVEF, and significant upregulation of natriuretic peptide type A (Nppa) and natriuretic peptide type B (Nppb) gene expression (Figures 1C–G).

**Figure 2:**
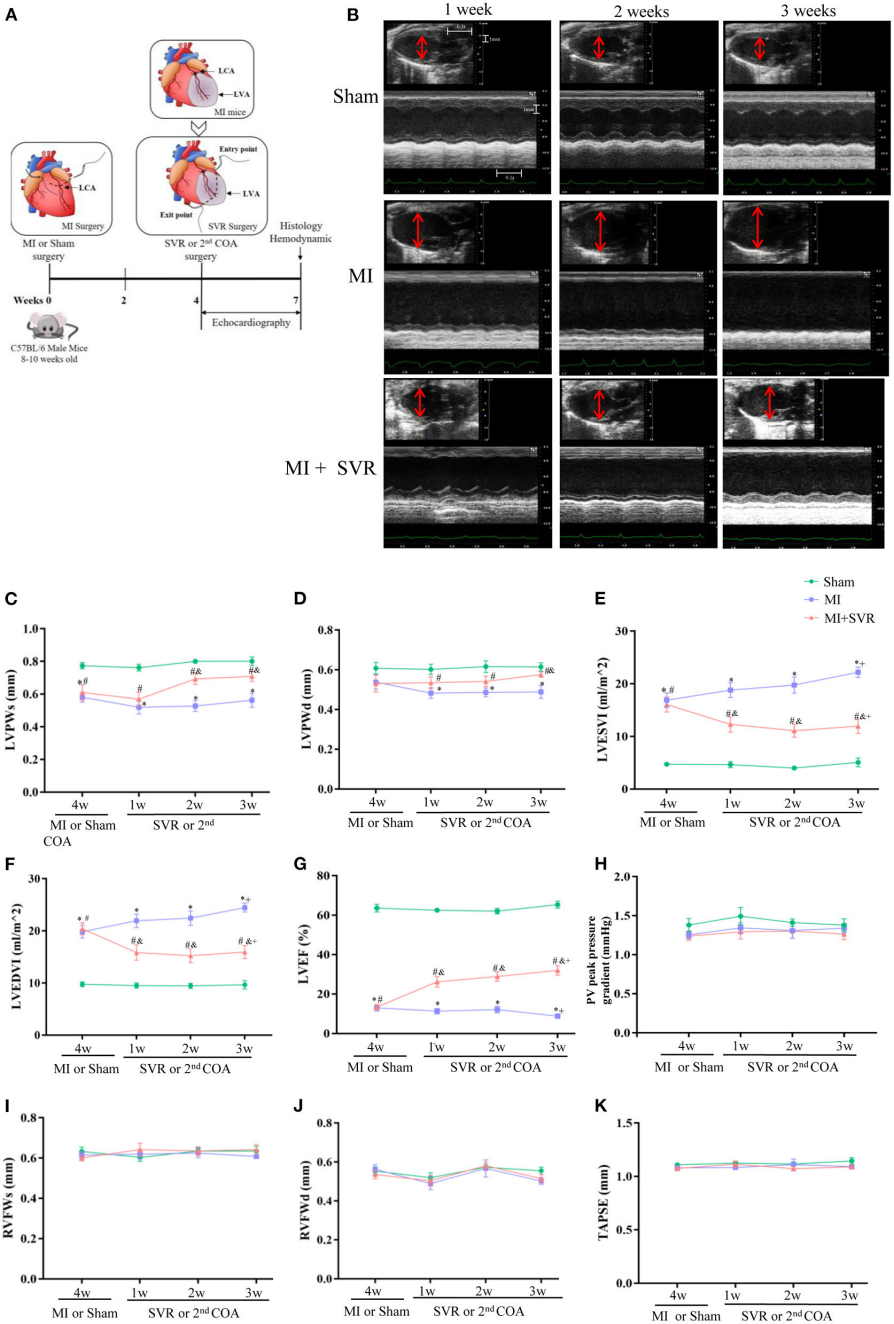
Effects of SVR surgery on remodeling and function of LV and RV evaluated by a time-course monitoring of Echocardiography from 1 to 3 weeks after SVR or 2nd chest-opening alone. **(A)** Schematic of MI and SVR mouse model. **(B)** Representative recordings of echocardiographic images of LV. The red lines indicate LV diameter. **(C)** LV systolic posterior wall thickness (LVPWs). **(D)** LV diastolic posterior wall thickness (LVPWd). **(E)** LV end systolic volume index (LVESVI). **(F)** LV end diastolic volume index (LVEDVI). **(G)** LV ejection fraction (LVEF). **(H)** The pulmonary valve (PV) peak pressure gradient. **(I)** RV systolic free wall thickness (RVFWs). **(J)** RV diastolic free wall thickness (RVFWd). For C-J, n=10 in each group. **(K)** Tricuspid annular plane systolic excursion (TAPSE), *n* = 5 in each group. *P < 0.05 between MI group and sham group; ^#^*P* < 0.05 between MI+SVR group and sham group; ^&^*P* < 0.05 between MI+SVR group and. MI group. ^+^*P*<0.05 vs. the initial value at 4 weeks after MI. MI, myocardial infarction; SVR, surgical ventricular reconstruction; LCA, left coronary artery; LVA, left ventricular aneurysm; COA, chest-opening alone; LV, left ventricle; RV, right ventricle; Data are means ± SEM.

Four weeks after MI, half of the mice with MI underwent SVR (Figure 2A). A time-course monitoring of echocardiography was performed in Sham, MI and MI + SVR groups (Figure 2B). The results indicated that the baseline data were similar between the MI and MI + SVR groups at 4 weeks after MI (Figures 2C–K), but that the LV posterior wall thickness exhibited a modest increase in SVR-treated mice but no significant change in untreated mice with MI (Figures 2B–D). Furthermore, the LV volume decreased and systolic function index of LVEF increased in a time-dependent manner in SVR-treated mice, whereas there was a gradual increase in LV volume and a decrease in LVEF in the untreated MI group (Figures 2B,E–G). However, no significant effect of SVR on right ventricular dimension and function was noted, as reflected by the pressure gradient across the pulmonary valve (PV maximum pressure), right ventricular free wall thickness, and TAPSE (Figures 2H–K).

To determine how long the SVR-induced improvement of LV remodeling persisted, we extended the observation period to 8 weeks after SVR. No significant changes in LV volume or LVEF were noted at 8 weeks after SVR compared to 3 weeks after SVR (Supplementary Figures 1A–E).

### SVR Improves Both the Systolic and Diastolic Function of Mice With HF After MI

**Figure 3:**
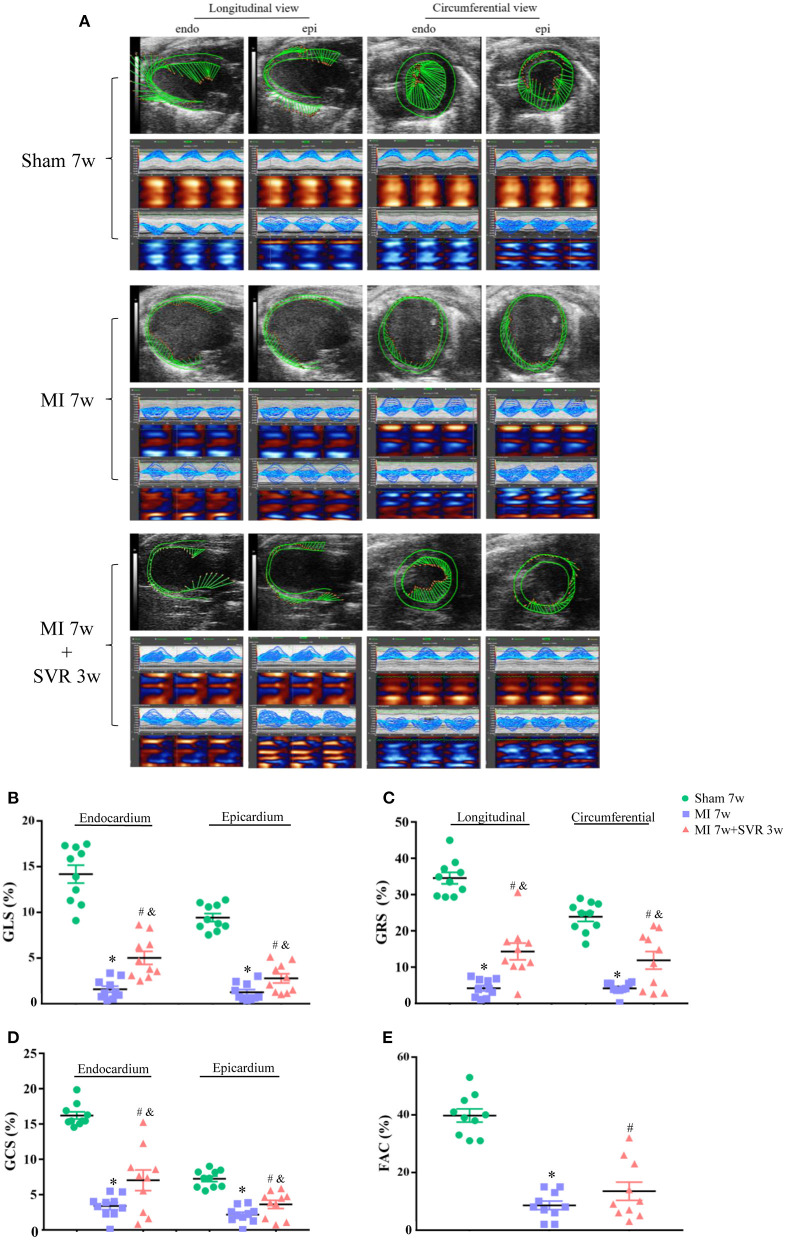
Effects of SVR on myocardial deformation of LV measured by speckle-tracking echocardiography at 3 weeks after SVR surgery. **(A)** Representative images of longitudinal and circumferential views of LV in each group. **(B)** Global longitudinal strain (GLS) in endocardium and epicardium. **(C)** Global radial strain (GRS) at longitudinal and circumferential view, respectively. **(D)** Global circumferential strain (GCS) in endocardium and epicardium. **(E)** Fractional area change (FAC). For B-E, *n* = 10 in each group. *P < 0.05 vs. sham 7w group; ^#^*P* < 0.05 vs. sham 7w group; ^&^*P* < 0.05 vs. MI 7w group. Data are means ± SEM. All the strain values were negative, indicating the direction of myocardial motion. To facilitate comparison, we used the absolute values in each group to plot. MI, myocardial infarction; SVR, surgical ventricular reconstruction; LV, left ventricle.

Speckle-tracking of echocardiography and invasive LV hemodynamic analysis were used to evaluate heart function 3 weeks after SVR. As shown in Figure 3A, myocardial motion in the sham group showed spiral morphology with a strong strain capacity in both the longitudinal and circumferential views, whereas in the MI group, the myocardial motion trajectory was irregular, and the strain capacity was weak. In contrast, mice with MI subjected to SVR had a much better myocardial trajectory and strain capacity than untreated mice. Quantitative analysis showed that the GLS, GCS, and GRS of the LV were markedly impaired in both the endocardium and epicardium in the MI group, while SVR reversed these trends (Figures 3B–D). Similarly, the LV FAC was also altered in the MI group, whereas SVR reversed this change to a certain extent (Figure 3E). These results collectively indicate that LV systolic function improves in response to SVR.

**Figure 4:**
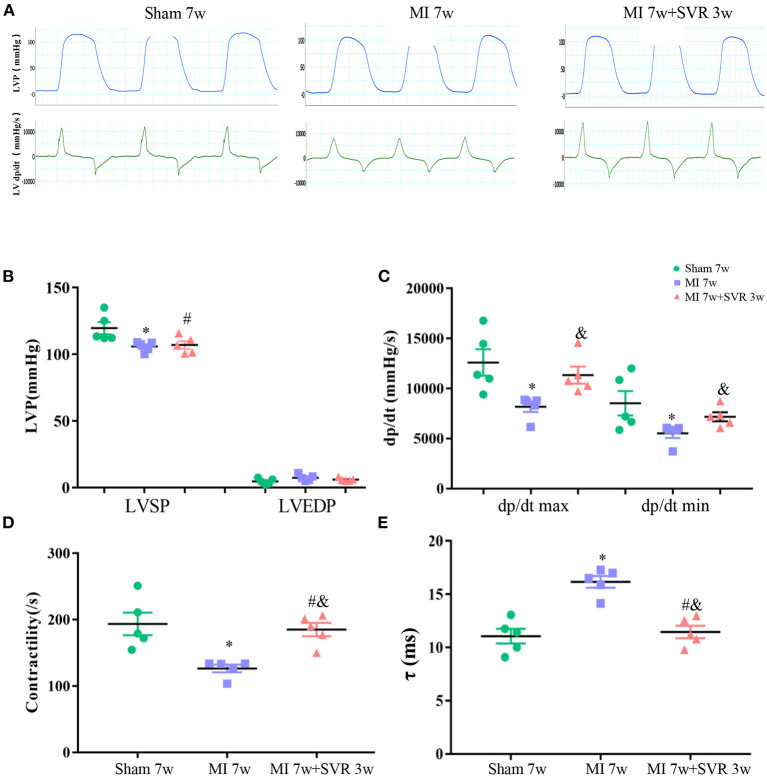
Effects of SVR on systolic and diastolic function of LV evaluated by invasive LV hemodynamics at 3 weeks after SVR surgery. **(A)** Representative pressure curve recordings of LV pressure and the rate of change of LV pressure. **(B)** LV systolic pressure (LVSP) and end-diastolic pressure (LVEDP). **(C)** Maximum rising rate of the LV pressure (dp/dt max) and maximum descending rate of the LV pressure (dp/dt min). **(D)** LV contractility. **(E)** Exponential time constant of relaxation (τ). For B-E, *n* = 5 in each group. *P < 0.05 vs. sham 7w group; ^#^*P* < 0.05 vs. sham 7w group; ^&^*P* < 0.05 vs. MI 7w group. Data are means ± SEM. MI, myocardial infarction; SVR, surgical ventricular reconstruction; LV, left ventricle.

We further analyzed the strain in 6 myocardium segments on the longitudinal and circumferential plane of the LV (Supplementary Figures 2A–H). The strain of the myocardium segments in the MI group was significantly impaired, which was reversed by SVR (Supplementary Figure 2A). Quantitative analysis showed that the radial strain of almost all segments on the longitudinal axis improved significantly after SVR, except the posterior and anterior apexes, which were the area of the LVA (Supplementary Figure 2C). SVR significantly improved the longitudinal strain of the endocardium and epicardium in posterior middle segment, as well as the longitudinal strain of the epicardium in anterior middle segment (Supplementary Figures 2D,E). The strain variation of the circumferential segments was not as uniform as that of the longitudinal segments. SVR markedly improved the radial strain in posterior wall and inferior free wall, the endocardial strain in anterior free wall and lateral wall, and the epicardium strain in posterior wall segment (Supplementary Figures 2F–H). LV hemodynamic analysis revealed that the mice with MI had a lower LV systolic pressure (LVSP), a lower maximum change rate of the LV pressure (LV dp/dt max and dp/dt min), lower LV contractility and a longer LV exponential relaxation time constant (τ) than in the sham group (Figures 4A–E), while the SVR group had a larger LV dp/dt max, a larger dp/dt min, greater LV contractility and a shorter τ than untreated mice with MI (Figures 4C–E). These results suggest that SVR can improve both LV systolic and diastolic function.

To further consolidate the beneficial effect of SVR on LV hemodynamic, we performed pressure-volume catheterization at 4 weeks after SVR, and noted a smaller end-systolic elastance and larger end-diastolic stiffness, LV end-diastolic pressure and τ in the MI group than in sham group, and these changes were partially reversed by SVR (Supplementary Figures 3A,B).

### SVR Attenuates MI-Induced Morphological and Histological Remodeling of the LV and Decreases the Expression of Molecular Markers of HF

**Figure 5:**
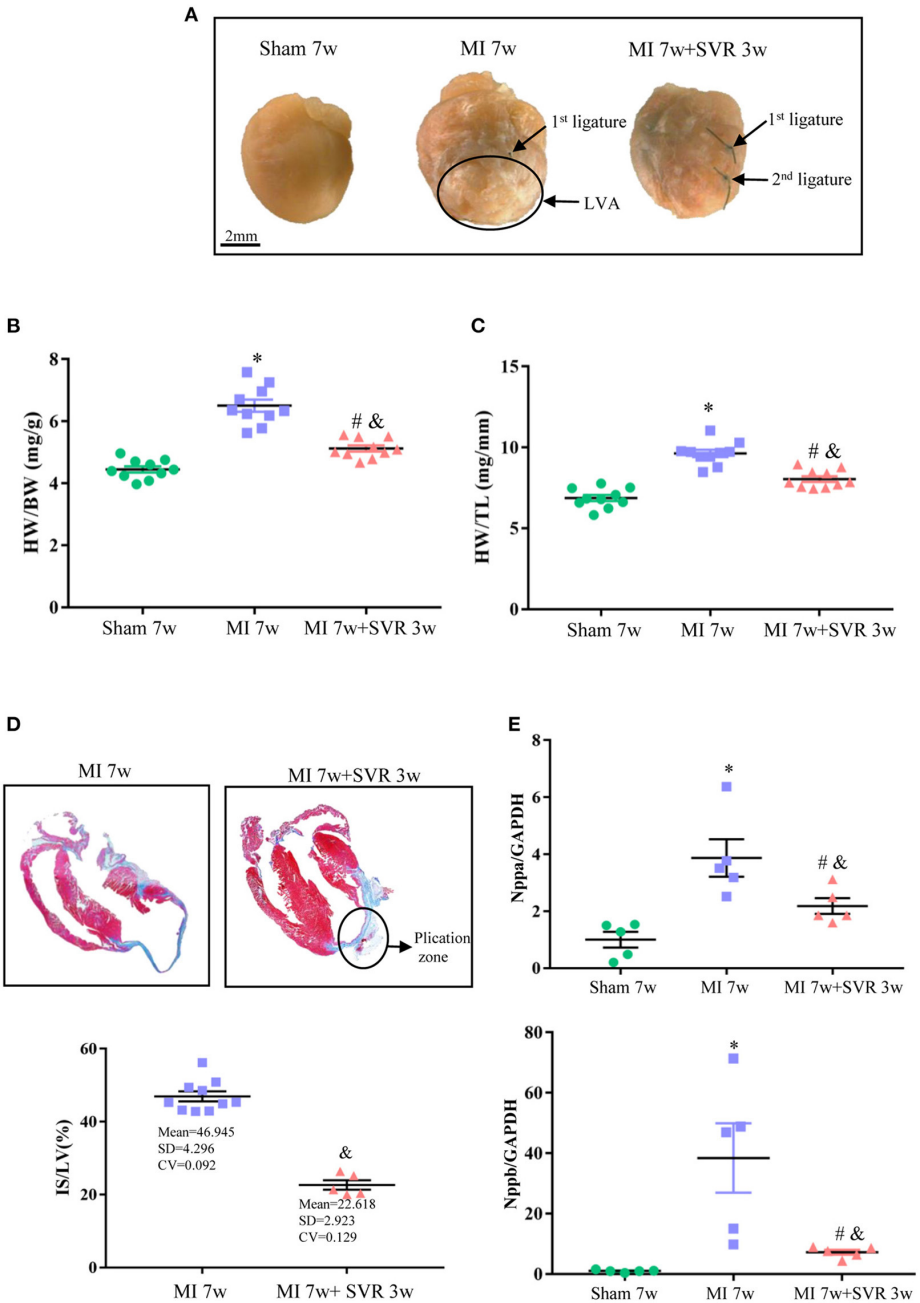
Effects of SVR on cardiac remodeling evaluated by histological analysis and biomarkers of heart failure at 3 weeks after SVR surgery. **(A)** Representative pictures of the whole heart in each group. Larger heart size and LV aneurysm are visible in MI 7w group rather than in MI 7W + SVR 3W group. **(B)** The heart weight to body weight ratio (HW/BW). **(C)** The heart weight to tibia length ratio (HW/TL). **(D)** Azan-Masson staining of the heart at longitudinal section in MI 7w and MI 7w + SVR 3w groups. The old infarct size (IS) was calculated by scar (blue staining) size/LV circumference size. **(E)** Real-time PCR for myocardial Nppa and Nppb gene expression. *P < 0.05 vs. sham 7w group; ^#^*P* < 0.05 vs. sham 7w group; ^&^*P* < 0.05 vs. MI 7w group. Data are means ± SEM. MI, myocardial infarction; SVR, surgical ventricular reconstruction; LV, left ventricle; Nppa, natriuretic peptide type A; Nppb, natriuretic peptide type B.

Three weeks after SVR, the hearts of SVR mice were markedly smaller than that of untreated mice with MI (Figure 5A). Quantitative analysis indicated that the SVR group had a smaller HW/ BW ratio and HW/ TL ratio, less myocardial fibrosis and lower expression of the Nppa and Nppb genes than untreated MI mice (Figures 5B–E). The typical longitudinal views of plication part are shown in Supplementary Figure 4.

### SVR Promotes Myocardial Regeneration and Angiogenesis in Mice After MI

**Figure 6:**
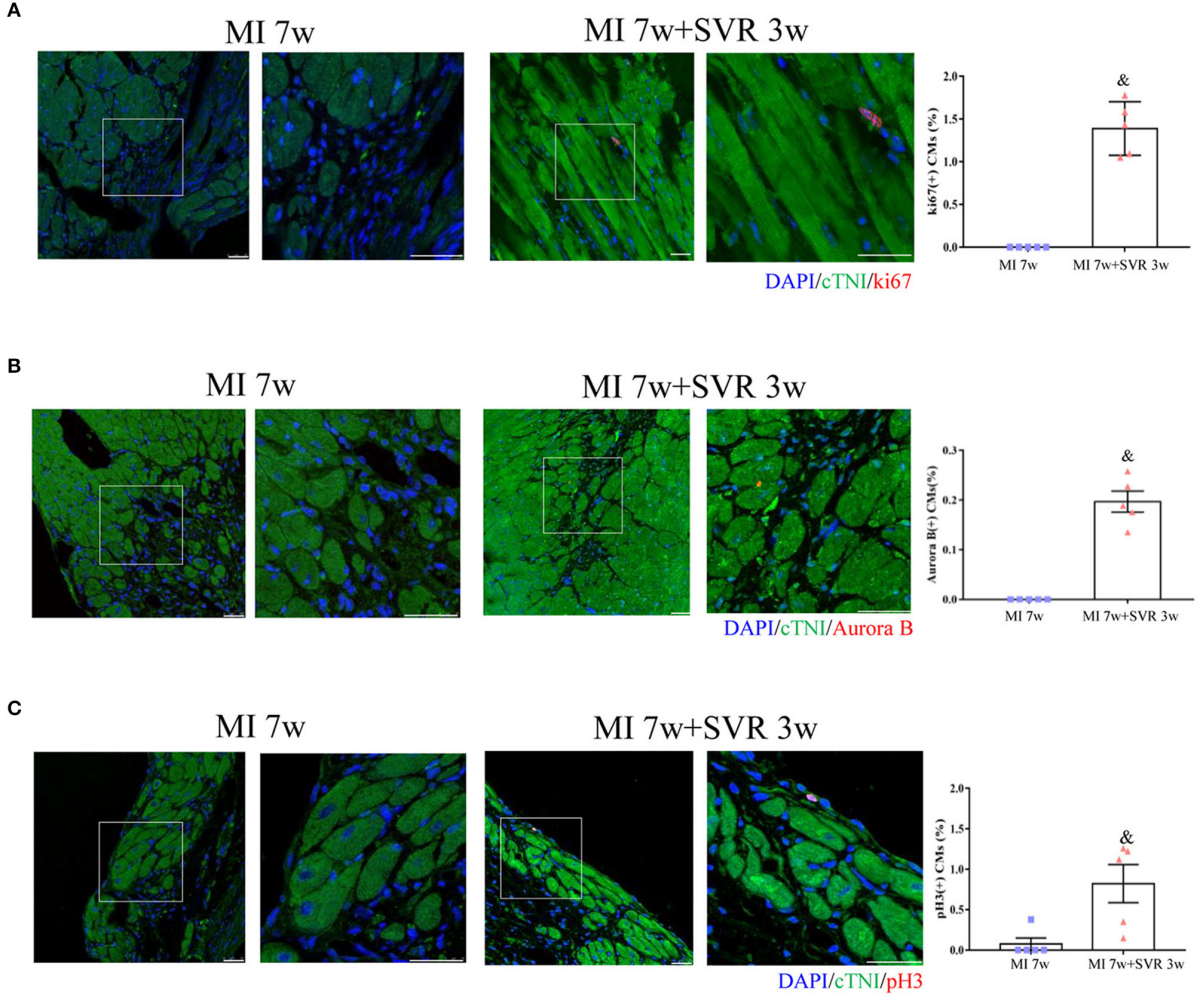
Effects of SVR on cardiomyocyte proliferation at the border or plication area at 3 weeks after 2nd surgery. **(A)** Ki67 positive-staining cardiomyocytes (CMs). **(B)** Aurora B positive-staining CMs. **(C)** pH3 positive-staining CMs. Bar = 25 um. ^&^*P* < 0.05 vs. MI 7w group. Data are means ± SEM. MI, myocardial infarction; SVR, surgical ventricular reconstruction. In each of the two side-by-side figures, the right figure is an enlarged view of the white box in the left figure.

**Figure 7:**
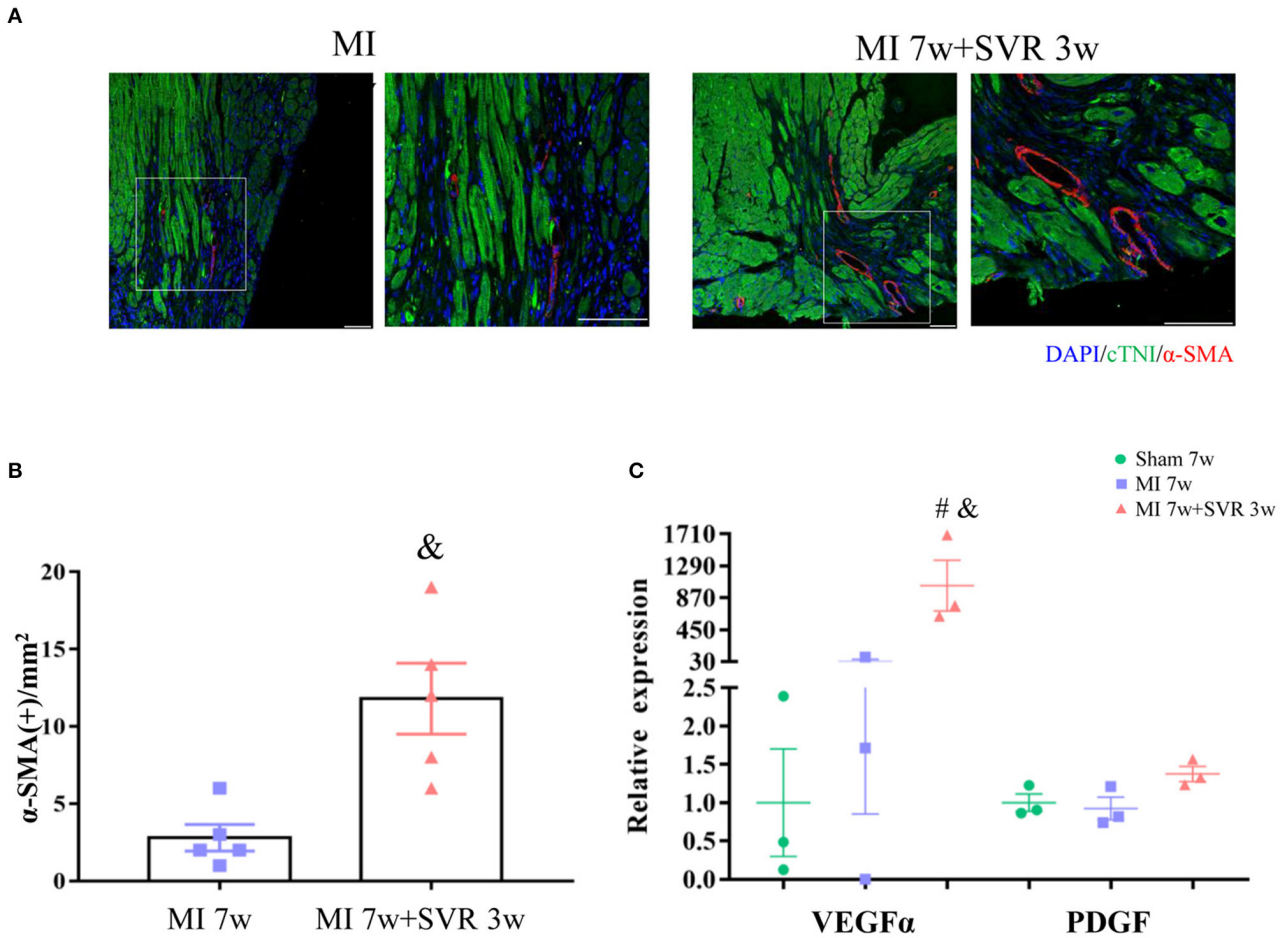
Effects of SVR on angiogenesis at the border or plication area at 3 weeks after 2nd surgery. **(A)** Representative pictures showing α-SMA positive staining vessels. **(B)** Semi-quantitation of α-SMA positive staining vessels. **(C)** Expression levels of vascular endothelial growth factor alpha (VEGFα) and platelet-derived growth factor (PDGF). Data are means ± SEM. ^&^*P* < 0.05 vs. MI 7w group, ^#^*P* < 0.05 vs. sham group. MI, myocardial infarction; SVR, surgical ventricular reconstruction. α-SMA, smooth muscle actin. In each of the two side-by-side figures, the right figure is an enlarged view of the white box in the left figure.

Seven weeks after MI, very few or no proliferating CMs were visible in the border zone of the heart, as indicated by staining for Ki67, Aurora B and pH3 (Figures 6A–C). In contrast, SVR led to a significant increase in CMs proliferation around the plication zone after 3 weeks, with the SVR group exhibiting a more than 2-fold increase in CM proliferation compared to the 7-week MI group (Figures 6A–C). These findings indicate that SVR enhanced the proliferative capacity of CMs. In addition, we found more alpha smooth muscle actin (α-SMA)-positive vessels and higher expression levels of vascular endothelial growth factor-α (VEGFα) around the plication zone in SVR-treated mice than in untreated mice, suggesting that SVR increases the vessel density in the heart after MI (Figures 7A–C).

## Discussion

We successfully performed SVR with a single diagonal linear suture and observed the following: (1) SVR resulted in a sustained reduction in LV volume and improvement in both systolic and diastolic function in mice with MI, LV aneurysm and dilatation; (2) the beneficial effects of SVR persisted for a long time without recurrent LV dilation, and SVR did not have adverse effects on the RV; and (3) SVR promoted CMs regeneration and angiogenesis around the plication zone. We believe this model is a helpful tool for clarifying the therapeutic effect of SVR and will pave the way for facilitating adjuvant therapy and cardiac regeneration to reverse HF.

Models of SVR have been established in rodents, sheep and rabbits (15, 26–30). The first animal model of SVR was generated using rats with MI by using a purse-string suture to plicate the LVA. The results showed that the reduction in LV remodeling did not last for longer than 4 weeks and even declined after 1 week (31), and similar results were reported in mice with MI (15). These results are inconsistent with research on epicardial ventricular reconstruction in sheep (28) and SVR with a purse-string suture in rabbits (30) and a recent clinical report with a follow-up of 7 years indicating that patients with dilated ischemic cardiomyopathy have favorable long-term outcomes after SVR (32). Different surgical procedures lead to different prognoses. Through the development of minimally invasive surgical procedures and percutaneous intervention, SVR has been modified from a cardiopulmonary bypass-supported large invasive surgery to a minimally invasive surgery combined with percutaneous intervention (33, 34), reducing operation mortality due to the low tolerance of patients with advanced HF to large-scale surgery. Fortunately, plication of the aneurysm with a single diagonal linear suture was sufficient to maintain the improvements in LV remodeling and heart function in our SVR mouse model for at least 8 weeks (possibly longer than 8 weeks).

The effect of SVR on LV systolic and diastolic function is controversial. Most clinical studies have shown that SVR can improve LV systolic function, in agreement with the finding of this study. Couperus et al. reported that SVR can worsen LV diastolic function in many HF patients with MI (10), while Castelvecchio et al. found that a large proportion of patients show improvement in LV diastolic function after SVR, with a small proportion continuing to deteriorate (35). In our current study, SVR significantly improved diastolic function in mice with MI, as evidenced by a shorter τ and a greater LV pressure descending rate. An early report indicated that the velocity of circumferential fiber shortening is a good prognostic index in SVR patients (36). Consistently, we noted that mice with MI subjected to SVR had a much better myocardial strain capacity.

Regarding the influence of SVR on right ventricular function, previous studies have reported that SVR may impair RV function possibly due to the existence of preoperative RV dysfunction and the elevation of LV filling pressure after surgery (37). In our study, neither preoperative RV dysfunction nor postoperative elevation of LV filling pressure occurred, and RV function was not affected by SVR in mice with MI. These results may illustrate that SVR itself does not affect right ventricular function but that right ventricular function may worsen after SVR only if it was already poor before surgery. In such cases, adjuvant therapy with optimal drugs is helpful for alleviating RV dysfunction.

We proved that SVR promoted cardiac regeneration around the plication zone, providing a basis for further adjunct therapy for regeneration. It is reasonable to speculate that a large aneurysm is an obstacle for myocardial regeneration, and environmental reforming by way of SVR would improve the efficiency of regenerative therapies. It has been reported that mechanical signaling in the microenvironment of CMs influences biochemical signaling and myocardial regeneration (38), while myocardial mechanics can be evaluated by strain analysis by speckle tracking (39). Our speckle-tracking echocardiography results indicated that myocardial strain was significantly improved in response to SVR, which may be a potential mechanism underlying the improvement in cardiac regeneration. Several reports have revealed that SVR can facilitate cardiac regenerative therapy and inhibit postoperative LV remodeling in rats (14, 40).

SVR for excessively dilated LV is to reduce LV volume by excluding the infarcted myocardial tissue, reshaping the LV and ameliorating HF. The findings of this study indicate that these changes occurred in our SVR mouse model and that further improvements in surgical procedures are required to achieve better reshaping the LV. Furthermore, it is noteworthy that we observed a reduction of LVESV immediately after SVR surgery, which is consistent with previous animal SVR studies (15, 28), but different from a previous report revealing no reduction in LVESV at 3 weeks after implantation of a transventricular reshaping device (41). We speculate that this discrepancy is due to the different surgical approach. By means of suture closure or epicardial catheter-based approach, a significant reduction of LVESV occurred immediately after SVR (15, 28), suggesting that direct isolating of the LVA could rapidly reduce LV volume and thus improve myocardial contractility, which is consistent with our findings. In addition, we noted that LVEDVI decreased by ~25% at one week after SVR in this study. Considering the incomplete plication of the infarct area, it is possible that the immediate reduction amplitude of EDV after SVR might be smaller than 25%, but the secondary improvements on hemodynamic and energy metabolism would further induce reversal remodeling of LV. According to Laplace's law, the ventricular wall tension is proportional to the radius of ventricular curvature, and ligation of the infarct area by diagonal sutures can reduce the LV volume, which in turn reduces the ventricular wall tension and myocardial oxygen consumption to improve the efficiency of cardiac work, and eventually promotes further reversal of LV remodeling (6, 42).

However, there are still some limitations to this study. First of all, our modified SVR approach is currently performed primarily in mice, and while the findings are significant and provide a new approach for basic research on SVR, it will take some time to expand to other mammals. Moreover, because folded LVA partially bulges outward due to intraventricular pressure, while the part without plication remains a larger outward expansion, thus it is impossible to cause a uniform outward expansion of the whole LVA by a single suture procedure. However, the tension of the oblique suture ensures that the area through which the suture passes is folded, which largely decreased the dilated LV. It was reported a high incidence of arrhythmias in patients treated with SVR through a double purse string (43, 44), suggesting complicated procedure is not necessarily better than a simple one. Second, cardiac regeneration is a complex subject. Although our study preliminarily confirmed that SVR surgery itself may provide an environment conductive to cardiac regeneration, stronger evidence, including core pathways and molecules, needs to be further explored, which is also the focus of our work in the future.

## Conclusion

Our modified SVR can reduce the LV volume, alleviate myocardial fibrosis, improve both systolic and diastolic function and promote myocardial regeneration in mice with MI and large aneurysms, and these beneficial effects are sustained. This model may facilitate basic research on the reversion of cardiac remodeling, cardiac regeneration and its mechanisms.

## Data Availability Statement

The original contributions presented in the study are included in the article/Supplementary Material, further inquiries can be directed to the corresponding authors.

## Ethics Statement

The animal study was reviewed and approved by the Ethical Committee of Nanfang Hospital, Southern Medical University.

## Author Contributions

YL, HL, and SM conceived and designed the study. SM and JY performed the experiments and wrote the manuscript. YL and HL reviewed and revised the manuscript. All authors analyzed, interpreted the data, and made important contribution to this work.

## Funding

This work was supported by grant from the Joint Funds of the National Natural Science Foundation of China (U1908205 to YL), the National Natural Science Foundation of China (82170278 to YL), the National Natural Science Foundation of China (82100407 to HL), the Project funded by China Postdoctoral Science Foundation (2021M690074 to HL).

## Conflict of Interest

The authors declare that the research was conducted in the absence of any commercial or financial relationships that could be construed as a potential conflict of interest.

## Publisher's Note

All claims expressed in this article are solely those of the authors and do not necessarily represent those of their affiliated organizations, or those of the publisher, the editors and the reviewers. Any product that may be evaluated in this article, or claim that may be made by its manufacturer, is not guaranteed or endorsed by the publisher.
