# Supplementary material for: A Modified Surgical Ventricular Reconstruction in Post-infarction Mice Persistently Alleviates Heart Failure and Improves Cardiac Regeneration
Source: Front Cardiovasc Med. 2021 Dec 24;8:789493. doi: 10.3389/fcvm.2021.789493 (PMC8740235; doi:10.3389/fcvm.2021.789493)
Supplement: Supplementary file 1 [file Data_Sheet_1.docx]

Supplementary Material

**Table S1. Sequences of primers for Real-time-PCR**

| Transcripts | Forward primer (5’–3’) | Reverse primer (5’–3’) | bps |
| --- | --- | --- | --- |
| Nppa (mouse) | GGCTCCTTCTCCATCACCAA | TGTTATCTTCGGTACCG | 420 |
| Nppb (mouse) | GAGGTCACTCCTATCCTCTGG | GCCATTTCCTCCGACTTTTCTC | 100 |
| GAPDH (mouse) | ATGTGTCCGTCGTGGATCTGA | TTGCTGTTGAAGTCGCAGGAG | 151 |
| VEGFa (mouse) | CTGCCGTCCGATTGAGACC | CCCCTCCTTGTACCACTGTC | 233 |
| PDGF (mouse) | TCTGCTGGGAACAACTCAACA | GTGAGACACCTCATCAGGGTAT | 157 |


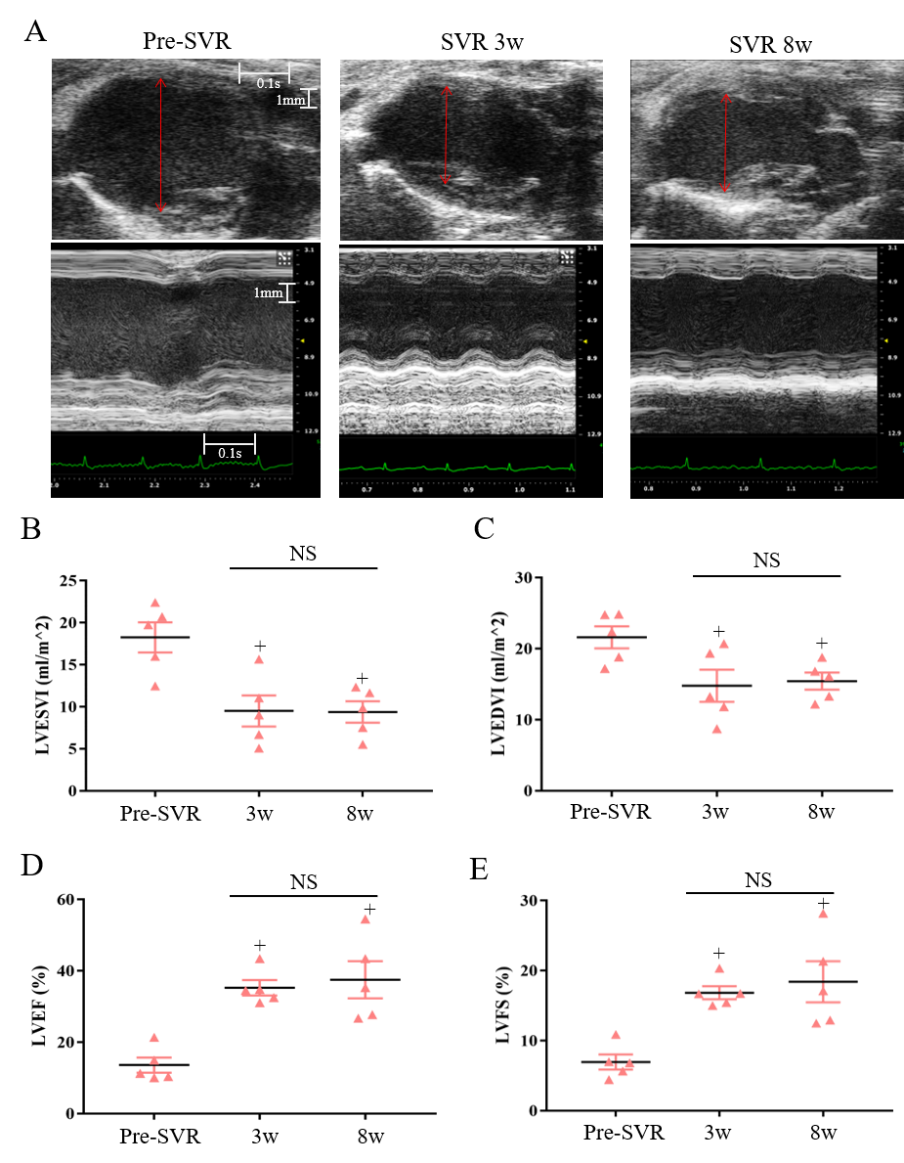


**Figure S1.** **Echocardiographic parameters in mice before SVR surgery or at 3 or 8 weeks after SVR surgery.** **(A)** Representative recordings of echocardiographic images of LV. The red lines indicate LV diameter. The white lines and corresponding arrows refer to the anterior wall (AW) or posterior wall (PW). **(B)** LV end systolic volume index (LVESVI). **(C)** LV end diastolic volume index (LVEDVI). **(D)** LV ejection fraction (LVEF). **(E)** LV fractional shortening (LVFS). n=5 in each group. ^+^ *P*＜0.05 vs. pre-SVR; NS, no statistical significance; Data are means ± SEM. LV*,* Left ventricle; SVR, surgical ventricular reconstruction.


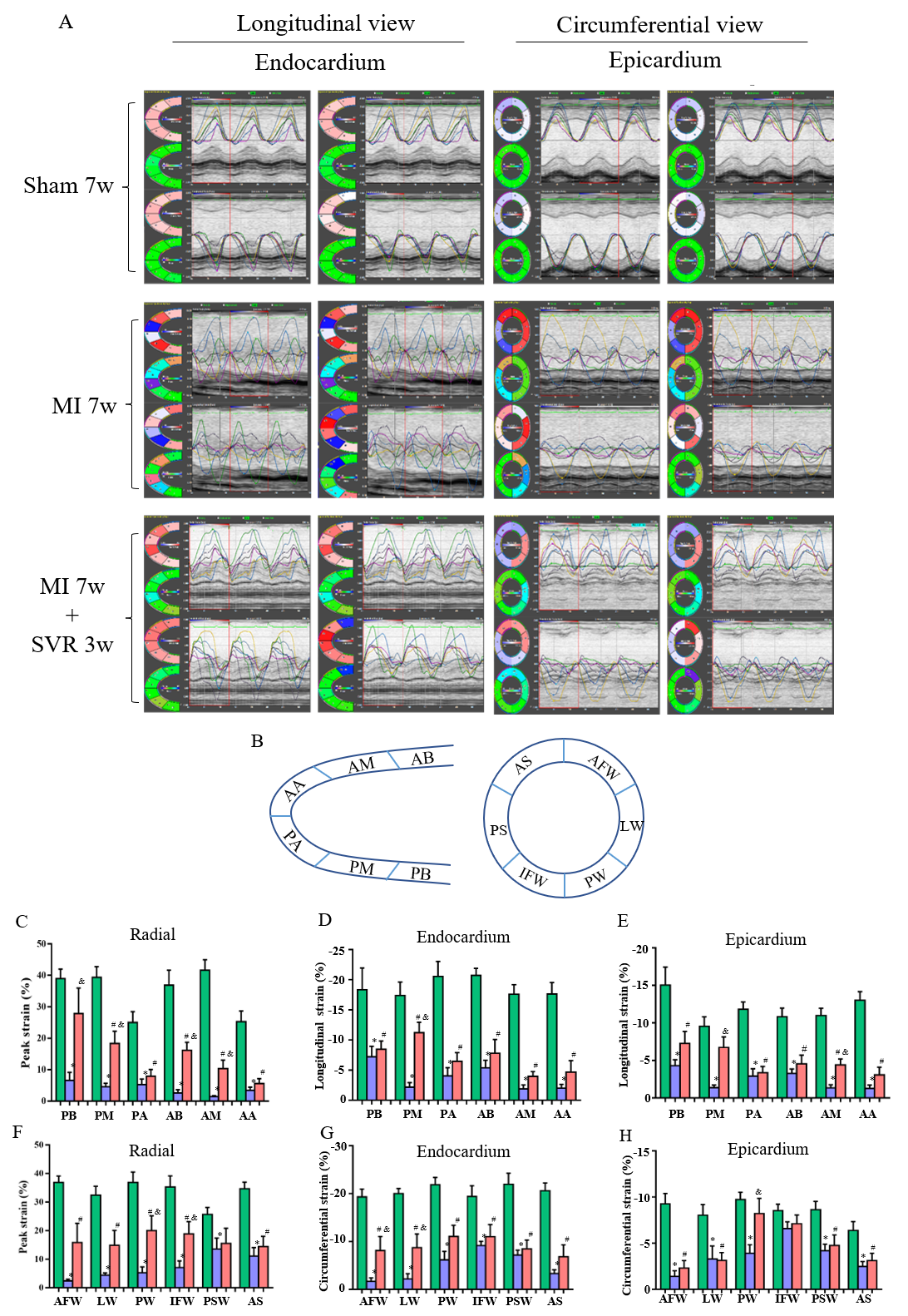


**Figure S2.** **Myocardial segmental strains of LV measured by speckle-tracking echocardiography at 3 weeks after SVR surgery. (A)** Representative images of longitudinal and circumferential views of LV in each group. **(B)** Schematic diagram of longitudinal and circumferential planes for location of myocardial segments. **(C)** Radial strains of 6 myocardial segments on the longitudinal axis. **(D)** The longitudinal strains in endocardium of 6 myocardial segments on the longitudinal axis. **(E)** The longitudinal strains in epicardium of 6 myocardial segments on the longitudinal axis. **(F)** Radial strains of 6 myocardial segments on the circumferential axis. **(G)** The longitudinal strains in endocardium of 6 myocardial segments on the circumferential axis. **(H)** The longitudinal strains in epicardium of 6 myocardial segments on the circumferential axis. For C-H, n=10 in each group. ^*^*P* < 0.05 vs. sham 7w group; ^#^*P* < 0.05 vs. sham 7w group; ^&^ *P* < 0.05 vs. MI 7w group. Data are means ± SEM. MI, myocardial infarction; SVR, surgical ventricular reconstruction; LV, left ventricle. AB, anterior base; AM, anterior middle segment; AA, anterior apex; PA, posterior apex; PM, posterior middle segment; PB, posterior base; AFW, anterior free wall; LW, lateral wall; PW, posterior wall; IFW, inferior free wall; PS, posterior septal wall; AS, anterior septum;


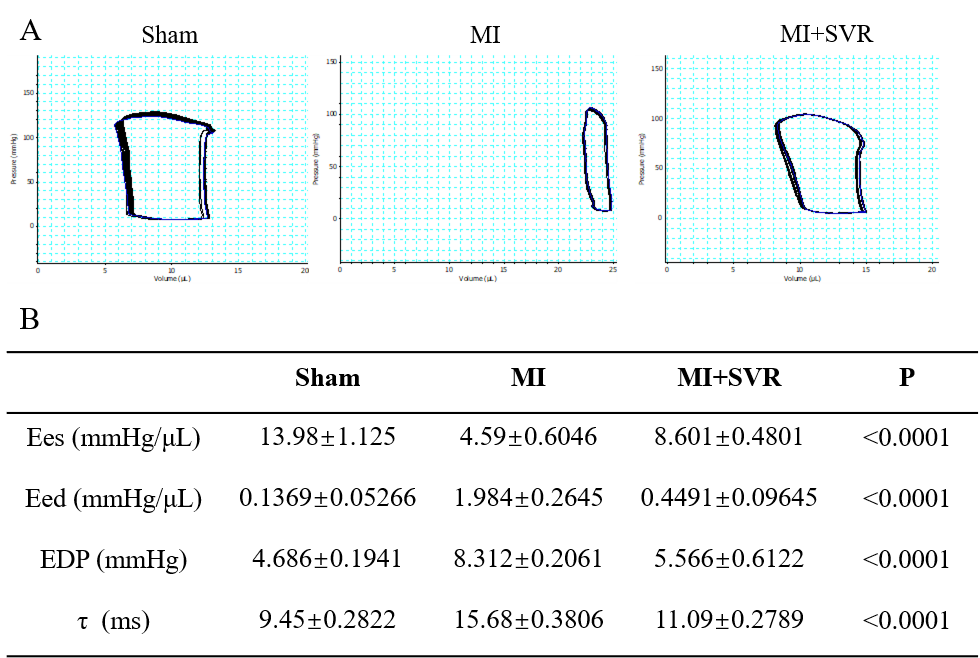


**Figure S3.** **Systolic and diastolic function of LV evaluated by PV loop** **at 4 weeks after SVR surgery. (A)** Representative images of each group. **(B)** The results of end-systolic elastance (Ees), end-diastolic stiffness (Eed), end-diastolic pressure (EDP), and exponential relaxation time constant (τ) among three groups. n=5 in each group. MI, myocardial infarction; SVR, surgical ventricular reconstruction, PV, pressure-volume.


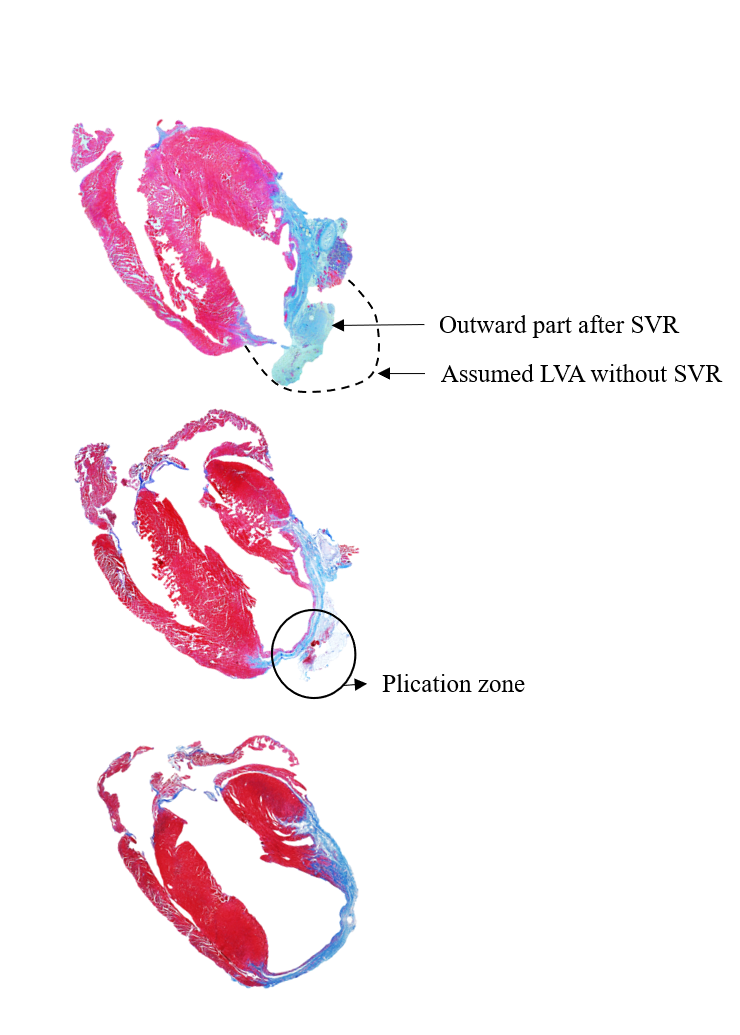


**Figure S4. Serial longitudinal sections of heart at 3 weeks after SVR.** LVA, left ventricular aneurysm; SVR, surgical ventricular reconstruction.

**Legend for Video S1**

**MI model:** Myocardial infarction surgery was performed on adult male C57BL/6 mice. The specific steps were as follows: (1) mice were anaesthetized with a mixture of xylazine and ketamine through intraperitoneal injection; (2) after confirming the anesthesia, keep the mouse in supine position by fixing the incisors with a suture and fixing the limbs; (3) clamp the tongue gently with forceps and then elevate the mandible with spatula to expose the glottis; (4) direct insert the trachea cannula and connect the ventilator; (5) apply hair removal cream to the chest area and connect the ECG machine as shown in the picture on the upper-right while waiting for the cream to take effect; (6) remove the hair from the chest and disinfect the expose skin with 75% alcohol; (7) make an incision and separate the skin; (8) blunt dissection of the muscular layer between the 3^rd^ and 4^th^ intercostals and expose the heart; (9) determine the entry point, around the left coronary artery at 2 mm from the tip of the left atrium, and ligature vessel using 8-0 nylon stitch; (10) make the double knot and then cut off the thread; (11) Suture the muscles and skin using 5-0 nylon stitch; (12) disinfect the skin again with 75% alcohol. Echocardiography was performed four weeks after myocardial infarction to confirm the formation of left ventricular aneurysm and significant enlargement of the left ventricle. Surgical ventricular reconstruction was performed after all parameters were determined.

**SVR surgery:** The specific steps were as follows: (1) anesthesia, fixation, endotracheal intubation, hair removal and incision were as described above; (2) blunt dissection of the muscular layer between the 4^th^ and 5^th^ intercostals, separate the adhesion between the heart and chest, and expose the left ventricular aneurysm; (3) determine the entry point, around the upper-left of the ligation knot remained by MI surgery, and the exit point, around the right-side of cardiac apex; (4) plicate the aneurysm with double-knot (it is important to make a surgical knot rather than a slippy knot) by a single-linear suture with a 6-0 nylon stitch without piercing the aneurysm; (5) after confirming no persistent bleeding, close the wound layer by layer and disinfect the skin as described above.
